# Supplementary material for: ProteinShader: illustrative rendering of macromolecules
Source: BMC Struct Biol. 2009 Mar 30;9:19. doi: 10.1186/1472-6807-9-19 (PMC2672931; doi:10.1186/1472-6807-9-19)
Supplement: Additional file 1 — ProteinShader program without source code. This compressed file contains the complete ProteinShader program including associated libraries, but no source code. A README.txt file gives an overview of the ProteinShader distribution, and the index.html file in the help subdirectory has directions on getting started with the program as well as a set of tutorials. [file 1472-6807-9-19-S1.zip › ProteinShader-beta-0_9_4-binary/help/tutorials/3POR.html]

ProteinShader Tutorial: 3POR


# ProteinShader Tutorial


♦ Home


♦ Download


♦ Getting Started

Choose a Link:

Installation

Running

Troubleshooting

PDB Files


♦ Tutorials

Choose a Link:

List of Proteins

Style Options

Saving Images


♦ General Controls

Choose a Link:

Menu Bar

Keyboard Shortcuts

Mouse Motion

Motion Panel

Antialiasing


♦ Cartoon Controls

Choose a Link:

Control Panels

Selection Panel

Radio Buttons

Decorations Panel

Adding Textures

Color Panel

Visibility Panel

Side Chains Panel


♦ Atom Controls

Choose a Link:

Control Panels

Selection Panel

Radio Buttons

Color Panel

Visibility Panel

Scale Panel

Tiling Panel


♦ Developers

Choose a Link:

Compiling

Running

Code Docs

Developer Links


## Porin: Transmembrane Beta-Barrel [PDB:3POR]

The porin protein is a transmembrane beta-barrel structure that serves as a
water-filled channel that allows nutrients and waste products to diffuse
through the outer membrane of gram-negative bacteria
[1, 2]. The complete porin
protein is composed of three identical subunits, each of which has a functional
channel. The three-dimensional structure of porin from
*Rhodobacter capsulatus* has been solved by x-ray crystallography
[2, 3], and
can be loaded into the ProteinShader program by selecting Open from the
File menu and then using the
file chooser box to select the 3POR.pdb file.


## Cartoon-style displays with halftoning

When the porin subunit is first loaded, it will be displayed as a
pen-and-ink style drawing of ribbons and tubes similar to what is shown in
Figure 1. To obtain something closer to the image in Figure 1A, first go to the
Decorations subpanel
and click on the Extra Lines checkbox. The extra lines that show up at regular
intervals along the ribbons and tubes mark the length of a ribbon or tube
that corresponds to an individual amino acid. Now click on the β-Strands
radio button to select all beta-strands, and select Hatching 1 from the
Bend Texture menu. The bend texture is the pattern that is applied to the
middle of each ribbon segment that corresponds to an amino acid, and the
intensity of the texture is scaled according to how bent the ribbon segment is.

To de-emphasize the loop regions, switch from the
Decorations subpanel
to the
Cartoon Visibility subpanel
and click on the Loops radio button before pressing the Translucent button.
The degree of translucency can be adjusted with the slider control below
the button. The semi-transparent loops stand out best against a white
background, so if the background is currently another color, use the
Background menu
above the canvas to select White. The orientation in Figure 1A, which shows
one end of the open channel, is the default view (Original or Front from the
Orientation menu
above the canvas). To obtain a side view of the beta-barrel as shown in
Figure 1B, select Bottom from the
Orientation menu,
and then drag the mouse across the canvas to make minor adjustments to the
orientation.


|  |  |
| --- | --- |
| **A** | **B** |

**Figure 1.** Ribbons and tubes style display of a subunit of porin.
(A) View of one open end of the beta-barrel structure.
(B) Side view of the beta-barrel structure.
The beta-strands are shown as ribbons, three small alpha-helices are shown
as tubes, and the loop regions are thin translucent ribbons.
The images were captured as described in the
Saving Images
section of the tutorials main page.


## Adding side chains to one loop

The porin channel is partially blocked by a loop region (plus a small
alpha-helix) between beta-strands 5 (G68-D74) and 6 (V118-A125)
[1, 4]. This loop,
which is labeled as loop 7 in the 3POR structure, fits inside the
channel, creating a narrow region region called the eyelet. The eyelet is
about 8 angstroms in diameter and 9 angstroms in length, and determines the
upper limit on the size of solute molecules that can diffuse through the
channel.

To visualize loop 7, assuming a starting point of Figure 1B, first select
Original from the
Orientation menu
above the canvas to get back to the orientation shown in Figure 1A. Then go to
the 
Cartoon Visibility subpanel
and select Loop 7 from the menu below the Loops radio button before clicking on
the Opaque button. Next go back to the
Decorations subpanel,
and with Loop 7 still selected, click the Plain button at the very bottom
of the subpanel. The default color for loop regions is gray, so switch
from the
Decorations subpanel
to the
Cartoon Color subpanel,
and with Loop 7 still selected, use the Chooser button to open a color chooser
dialog box, and select a shade of red. The image should look similar to
Figure 2A, although the exact orientation will need to be adjusted by dragging
across the canvas with the mouse.

To get a better sense of how much of the channel is filled by loop 7, its
amino acid side chains can be shown in a space filling style display as in
Figure 2B. First, use the menu at the top right of the Control Panel to
switch to the
Cartoon Side Chains subpanel.
After checking that Loop 7 is still selected, click on the Space Filling
button. By default, the spheres used in a space filling style display are
colored by atom type. To color them by amino acid type, switch to the
Atom Color subpanel,
select Loop 7 in the menu below the Loops radio button,
and then click on the Amino Acid button at the very bottom of the subpanel.
The image should now be very close to Figure 2B.


|  |  |
| --- | --- |
| **A** | **B** |

**Figure 2.** A loop region narrows the size of the porin channel.
(A) The same image as Figure 1A, except that loop 7 is shown in red, and the
protein has been tilted slightly to get a better view of the loop.
(B) Same as part A, but the amino acid side chains of loop 7 are shown
as a space filling style display. The side chains are colored by amino acid
type. The images were captured as described in the
Saving Images
section of the tutorials main page.


## References

1.
Brandon C, Tooze J: **Membrane Proteins.**
In *Introduction to Protein Structure.* 2nd edition.
New York: Garland Publishing; 1998: 223-250.

2.
Weiss MS, Schulz GE:
**Porin conformation in the absence of calcium.
Refined structure at 2.5 A resolution.**
*J Mol Biol* 1993, **231**: 817-824.

3.
**Porin PDB entry 3POR**
[http://www.rcsb.org/pdb/explore/explore.do?structureId=3POR]

4.
Weiss MS, Abele U, Weckesser J, Welte W, Schiltz E, Schulz GE:
**Molecular architecture and electrostatic properties of a bacterial porin.**
*Science* 1991, **254**: 1627-1630.


---

|  |  |  |  |
| --- | --- | --- | --- |
| Hosted by |  |  |  |

Webmaster: Joe Weber,
joe.weber AT alumni.duke.edu
  
Web site last updated: December 30th, 2008
  
© 2007–2008 Joseph R. Weber
